# Experimental Investigation on the Acoustic Insulation Properties of Filled Paper Honeycomb-Core Wallboards

**DOI:** 10.3390/biomimetics9090528

**Published:** 2024-09-01

**Authors:** Yiheng Song, Haixia Yang, Nanxing Zhu, Jinxiang Chen

**Affiliations:** 1 Key Laboratory of Concrete and Prestressed Concrete Structures of Ministry of Education, School of Civil Engineering, Southeast University, Nanjing 211189, China; utsongyih@gmail.com (Y.S.); yhxyanghaixia@outlook.com (H.Y.); zhunanxing1997@163.com (N.Z.); 2 School of Engineering, The University of Tokyo, Tokyo 113-8656, Japan

**Keywords:** sound insulation performance, sandwich plates, honeycomb structure, straw filling, reverberation chamber–anechoic chamber method

## Abstract

Honeycomb plates, due to their multi-cavity structure, exhibit excellent mechanical properties and sound insulation. Previous studies have demonstrated that altering the cell size and arrangement of honeycomb structures impacts their acoustic performance. Based on these findings, this study developed a wallboard structure with enhanced sound insulation by filling the cavities with paper fiber/cement facesheets and designing a stacked core structure. Through the reverberation chamber–anechoic chamber sound insulation experiment under 100–6300 Hz excitation and conducting orthogonal experiments from three dimensions, it was found that: (1) Compared to no filling, the filling with straw and glazed hollow bead can increase the sound transmission loss (*STL*) by more than 50% in the frequency bandwidth above 2000 Hz. This indicates that both types of fillings can significantly enhance the sound insulation performance of the honeycomb structure without a significant increase in economic costs. (2) The increase in paper fiber/cement facesheets improves the *STL* across the entire experimental bandwidth, with a maximum improvement exceeding 70%. This structural design not only offers superior sound insulation performance but also better suits practical engineering applications. (3) Increasing the number of core stacking units (from one to three), taking straw-filled paper honeycomb-core wallboards as an example, effectively increased the *STL* bandwidth. (4) This test enriches the application of honeycomb plates in sound insulation. Introducing fiber paper fiber/cement facesheets and eco-friendly, low-cost straw improves sound insulation and enhances the strength of honeycomb, making them more suitable for construction, particularly as non-load-bearing structures.

## 1. Introduction

In the field of architecture focused on environmental protection, energy conservation, and sustainable development, bionic structures have increasingly become a research hotspot. These structures draw inspiration from nature, incorporating the wisdom and design principles observed in the natural world [1,2,3,4]. One exemplary bionic structure is the honeycomb sandwich structure, which mimics the geometry of a beehive. Bees build their nests to resist external impacts and intrusions, such as wind. Additionally, because the nest houses a large number of bees and stored food, it requires effective heat dissipation and ventilation. Consequently, the honeycomb structure, refined through natural selection, not only exhibits superior mechanical properties such as high specific strength and stiffness [5,6,7] but also effectively reduces heat conduction and provides excellent thermal performance [8,9,10]. Additionally, it provides excellent sound insulation [11,12]. These characteristics make honeycomb plates a biomimetic structure that is widely studied and adopted in the industry.

The excellent sound insulation of honeycomb plates is mainly due to the multi-cavity arrangement on their two-dimensional plane [13]. Because sound waves are mechanical waves, they need a medium to propagate, and the honeycomb structure effectively interrupts and disperses the propagation path of sound waves through its multi-cell geometry, reducing the transmission of sound. This multi-cavity layout makes the honeycomb plate an excellent sound insulation structure [14,15,16,17]. It is especially suitable for non-load-bearing wallboards in buildings, effectively addressing noise pollution issues [18]. In the study of the acoustic insulation performance of honeycomb structures, it has been found that the cell size and cell arrangement of honeycomb structures have impacts on their sound insulation performance. For example, Yinmei Ge et al. researched and summarized that the geometry of the honeycomb structure can cause sound waves to scatter and reflect multiple times [19,20]. And Jae-Deok Jung et al. demonstrated that smaller honeycomb cell sizes and thicker cell walls improved sound insulation performance, especially at high frequencies [21]. Additionally, MP Arunkumar et al. observed that reducing the height of the honeycomb core to 20 mm and increasing the panel thickness to 3 mm improved sound insulation at low frequencies [22]. Furthermore, Saber Saffar found that a 1 × 1 cell arrangement at a 45° angle configuration exhibited the highest sound absorption coefficient, particularly in the 100 Hz to 500 Hz frequency range [23].

In order to further improve the sound insulation performance of honeycomb plates, it is possible to utilize their cavity structure fully by adding lightweight filling materials [24]. For example, plant fiber materials in nature, such as straw and flax, have a porous structure similar to a honeycomb structure, which can effectively absorb sound waves and are therefore considered ideal filling materials [25,26,27]. In addition, glazed hollow beads are widely recognized in the market for their microstructure and excellent sound insulation performance, making them one of the most popular sound insulation materials available [28,29,30]. Foam materials are also extensively used for filling and sound insulation due to their flexibility, diverse shapes, and superior sound insulation effects [31,32,33].

Given these possibilities, this study selects the most commonly used paper honeycomb available on the market and conducts filling experiments while keeping the honeycomb structure fixed. The research examines the effects of filling materials, facesheet configurations, and core types on the sound insulation of the paper honeycomb structure. By investigating these factors, this study aims to provide comprehensive insights into optimizing the material structure and configuration to achieve superior acoustic performance in paper honeycomb-core wallboards. Combined with the existing research results on filled honeycomb plates having better mechanical performance [34], the findings of this study will contribute to the development of lightweight and high-strength honeycomb structures with enhanced sound insulation capabilities, making them more effective for use in a variety of construction applications where noise reduction is a critical concern.

## 2. Sample Preparation and Experimental Methods

### 2.1. Model Design and Preparation

#### 2.1.1. Model Design

**Figure 1:**
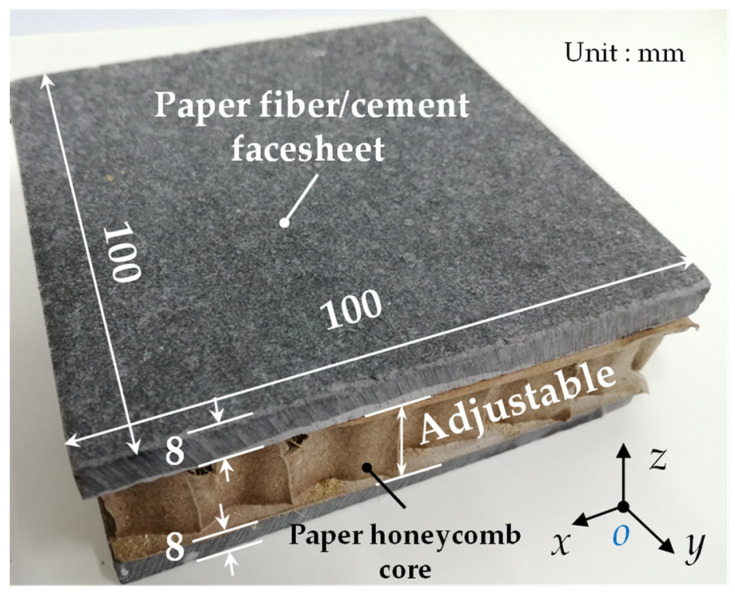
The filled paper honeycomb-core wallboards proposed in this paper.

The filled paper honeycomb-core wallboard (FHW) proposed in this paper is mainly composed of upper and lower paper fiber/cement facesheets and a filled paper honeycomb-core in the middle. The specific external contour dimensions are shown in Figure 1. The facesheets were made of paper fiber-reinforced cement board (Fet Building Materials and Technology Co., Ltd., Ningbo, China). The length of the paper fibers used in these facesheets ranged from 2.5 to 3 mm, the doping was 10.0 vol%, and the density was 1.3 g·m^−3^. And the modulus of elasticity and flexural strength of the material, in its air-dry state, were approximately 6.0 GPa and 15.0 MPa, respectively. The skeleton of the core was made of grade A kraftliner paper with a gram weight of 110 g·m^−2^ (Dongshan Paper Products Factory, Jurong, China). The facesheets and the core were bonded using a polyvinyl acetate emulsion (PVAc, EOC Polymers Co., Ltd., Shanghai, China) [35].

To explore sound insulation performance of the wallboard more comprehensively, this study conducted orthogonal experiments across three dimensions [36]: different filler materials (3 + 1 types, with 1 type being no filler), the presence or absence of facesheets (2 types), and types of core formation (3 types). The presence or absence of facesheets in the honeycomb structure is straightforward and will not be reiterated. Detailed explanations of the first two dimensions, involving different filler materials and types of core formation, are provided below.

(1) Filling materials

In this paper, three kinds of lightweight filling materials, namely straw, glazed hollow bead, and foam, were selected (Table 1). These materials were inserted into the multi-cavity with equal mass, ensuring the same apparent volume was maintained. As mentioned in Section 1, all three materials exhibit excellent sound insulation effects. Additionally, straw is sustainable and environmentally friendly, making it a natural choice for eco-friendly construction materials [37]. Glazed hollow beads can provide effective thermal insulation [28]. And foam is cost-effective and highly adaptable, easily filling cavities and providing effective sound insulation [38]. Each material offers unique advantages for sound insulation applications in construction.

Subsequently, the facesheets were pasted on the upper and lower surfaces to create the straw-filled, glazed hollow bead-filled, and foam-filled paper honeycomb wallboards, which are denoted as FHW_S_, FHW_G_, and FHW_F_, respectively. Meanwhile, in order to facilitate a comprehensive comparative analysis, paper honeycomb wallboards without any filler, denoted as FHW_E_, were utilized as the comparison object. This approach was employed to investigate the effect of the aforementioned different filler materials on the acoustic performance of the FHW.

(2) Core formation types

**Figure 2:**
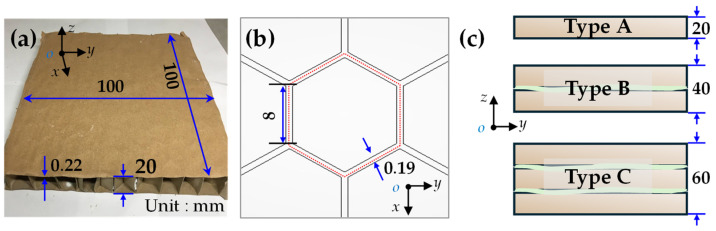
Core unit and three types. (**a**) External contour dimensions of the unit. (**b**) Honeycomb cell. (**c**) Schematic diagram of the three types.

The core employs a honeycomb structure. The core unit was designed according to the design principle of an optimal height-to-thickness ratio, with its external contour and honeycomb cell dimensions illustrated in Figure 2. The height of the core unit is 20 mm. Subsequently, three distinct core formation types were defined [39]. Based on the number of units included within each formation, they are classified as type A (containing 1 unit), type B (containing 2 units), and type C (containing 3 units).

#### 2.1.2. Sample Preparation

**Figure 3:**
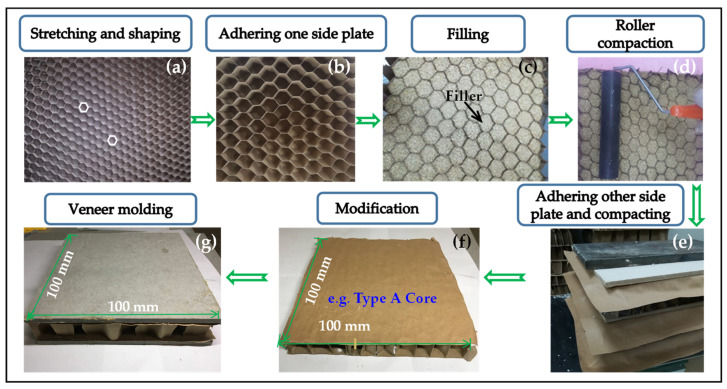
Preparation process of wallboard samples: from core arrangement to final assembly.

Figure 3 illustrates the preparation process for the specimens. First, as depicted in Figure 3a,b, the arrangement of the core unit framework is complete. Then, the filling material is selected, ensuring uniform filling and compaction (Figure 3c,d). This step eliminates the need for glue, thereby streamlining the process, reducing costs, and enhancing the environmental sustainability of the filled paper honeycomb structure [40]. Subsequently, the complete encapsulation of the unit is carried out (Figure 3e,f). Finally, paper fiber/cement facesheets are adhered to the surface of the core, thereby forming the final wallboard sample.

Afterwards, to minimize the impact of temperature and humidity on the test results, the specimens need to be conditioned in a constant temperature and humidity test chamber (AT-HW-1000, Anymet Instruments, Jinan, China) with a curing temperature of 23 °C and a relative humidity of 50% for a duration of 48 h. Following the curing process, each specimen is individually sealed in a sealed bag, taken out for testing, and each test is completed within a time frame of 5 min.

### 2.2. Sound Insulation Performance Test Method and Indicators

**Figure 4:**
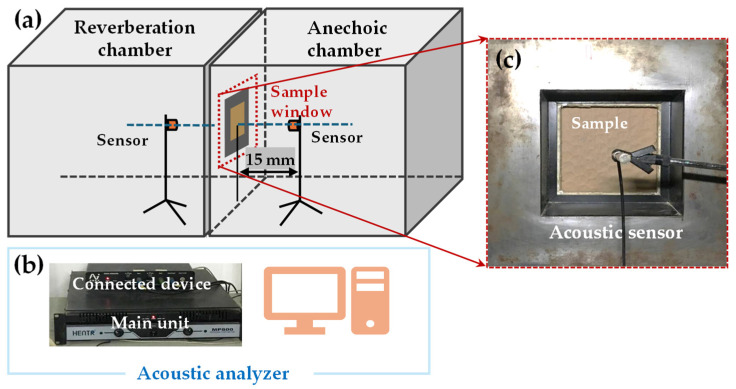
Acoustic test equipment and schematic diagram. (**a**) The schematic diagram of reverberation chamber–anechoic chamber. (**b**) Acoustic analyzer and processing device. (**c**) Side view of the sample window from the perspective of the anechoic chamber.

The experiment uses a dual-channel acoustic analysis testing system (BSWA VS302USB, Shengwang Electric Technology Co., Ltd., Beijing, China) to test the sound insulation performance (Figure 4) [41]. The experiment setup consists of a reverberation chamber and an anechoic chamber (Figure 4a), with a sample window (Figure 4c) positioned in the middle and acoustic sensors placed on both sides of the sample window, 15 cm away from the sample, with the symmetrical center located at the center of the sample. These sensors measure the acoustic signals in the reverberation chamber and the anechoic chamber, respectively.

The test method proceeds as follows: Initially, without installing the sample, white noise is emitted within the reverberation chamber. After a stable sound field has been established, sensors placed in the reverberation chamber and the anechoic chamber receive the sound signal, and the sound pressure level data is subsequently obtained through processing. Next, the sample is installed, and the sound pressure level test is conducted in the same manner. Finally, the sound transmission loss (*STL*) of the sample is calculated, which serves as the sound insulation performance index utilized in this paper. The calculation formula is as Equation (1):

(1) $$\mathrm{STL}=\;(L_{1}\;-\;L_{2})+(L_{1}^{\prime }-L_{2}^{\prime })$$

where *L*_1_ is the sound pressure level of the reverberation chamber when the sample is installed; *L*_2_ is the sound pressure of the anechoic chamber when the sample is installed; *L′*_1_ is the sound pressure of the reverberation chamber when the sample is not installed; and *L′*_2_ is the sound pressure of the anechoic chamber when the sample is not installed.

To enhance the clarity of the conversion and transmission of sound pressure signals, a detailed demonstration of the calculation process of the transfer function will be provided. First, Equations (2) and (3) are used to transform the time domain into the frequency domain. Then, based on Equation (4), the transfer function *H*(*f*) is derived.

(2) $$P_{in}\left(f\right)=F\left\{P_{in}\left(t\right)\right\}$$

(3) $$P_{out}\left(f\right)=F\left\{P_{out}\left(t\right)\right\}$$

(4) $$H\left(f\right)=\frac{P_{out}\left(f\right)}{P_{in}\left(f\right)}$$

where *f* is frequency, *t* is time, and F denotes the Fourier transform. *P*_in_ is the sound pressure signal on the reverberation chamber side, and *P*_out_ is the sound pressure signal on the anechoic chamber side.

The measurement is carried out according to ISO 10140-1, the standard for sound insulation measurement of buildings and building components [42]. Initially, a baseline measurement of the environment is conducted, and this involves measuring the natural attenuation of noise and the original sound pressure level in the environment without the presence of any sound insulation materials. Following this, the formal measurement is performed using an A-weighted network along with a pink background noise source set to a sound pressure level of 80 dB. The volume of the silent box is 1 × 1 × 1 m^3^. The sampling frequency for the sound is set to 48,000 Hz, with the extraction rate selection set to “Infinite” and the number of fast Fourier transform samples configured to 2048. The data measurement employs the commonly used 1/3 octave band method. Finally, the sound attenuation data of the sample is compared with the baseline test data to determine the actual sound attenuation effect of the sample. This comparison reveals the effectiveness of the sound insulation properties of the wallboards under investigation.

## 3. Results and Discussion

This section presents the differences and changing trends in the sound insulation performance for FHWs. This analysis is conducted under varying conditions, including honeycomb plates filled with different materials, the presence or absence of facesheets, and different core types (classified as type A, type B, and type C). By examining these specific conditions, the underlying factors that affect the sound insulation properties of the FHWs can be better understood and elucidated.

### 3.1. Influence of Filling Materials

**Figure 5:**
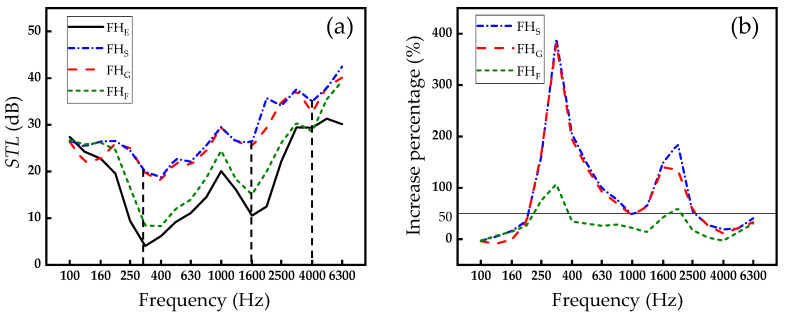
Sound insulation performance of FHs with different filling materials. (**a**) *STL*–frequency curve. (**b**) Improvement percentage–frequency curve.

Due to the relative ease associated with studying the paper honeycomb-core unit (FH), the initial focus is on analyzing the influence of various filling materials on the acoustic performance of FH. These filling materials include no filling, straw filling, glazed hollow bead filling, and foam filling, which are abbreviated as FH_E_, FH_S_, FH_G_, and FH_F_. The representative *STL*–frequency curves for different FHs with different fillings are depicted in Figure 5a. Overall, among FH_E_, FH_S_, FH_G_, and FH_F_, there are two pairs of similar phenomena, namely, the curves of FH_E_ and FH_F_ are close, as are the curves of FH_S_ and FH_G_. Specifically, in the initial stage, as the frequency increases, the *STL* of the honeycomb structure drops, and when the frequency reaches around 315 Hz (the first black dotted line), the *STL* reaches its minimum value. This is mainly because as the frequency of the sound wave gradually approaches the natural frequency of the honeycomb structure, a resonance effect occurs [43,44], i.e., the sound insulation performance of the honeycomb structure is minimal at its natural or resonance frequency because the internal vibrations of it reach their maximum amplitude at this frequency, greatly increasing the transmission efficiency of acoustic waves. Meanwhile, it can be observed that the rate of decline in *STL* for FH_E_ and FH_F_ is significantly higher than that for FH_S_ and FH_G_, indicating that FH_S_ and FH_G_ are less affected by the rapid attenuation of sound insulation caused by the resonance effect, and the sound insulation capacity of the plates remains very stable. When the sound wave frequency reaches 1600 Hz (the second black dotted line), it coincides with another natural frequency, causing another dip in *STL* due to resonance. And the sound insulation performance in other frequency regions is primarily governed by the mass law of the honeycomb structure. The mass law indicates that as the frequency increases, the honeycomb structure’s ability to block sound waves improves, resulting in an increase in *STL* with frequency [45,46]. When the incident sound wave frequency continues to increase, the sound wave striking the upper and lower paperboards of the honeycomb-core causes structural bending vibrations, leading to a decrease in *STL*, with a trough appearing around 4000 Hz (the third black-dotted line) [40]. And then the sound insulation performance is still governed by the mass law.

In order to better assess the impact of filling materials on the sound insulation performance of FHs, this section calculates the relative improvement of *STL*s of different filling materials compared to no filling material (FH_E_). The improvement percentage is used to identify frequency intervals where the increase in sound insulation capacity exceeds 50%. These intervals are then aggregated to obtain the bandwidth, which serves as the judgment criterion. It can be seen from Figure 5b that FH_S_ has the best sound insulation performance, and the bandwidth can reach 2310 Hz. This is followed by FH_G_ at 300 Hz and FH_F_ at 560 Hz. The superior performance of FH_S_ and FH_G_ can be attributed to the higher damping coefficients of these filling materials, which can more effectively absorb and dissipate the vibrational energy caused by sound waves, reducing the propagation of sound waves within the honeycomb structure [47]. And the smaller reduction in *STL* at the resonant frequencies of FH_S_ and FH_G_ can also be attributed to damping, which suppresses resonance and enhances sound insulation performance by reducing peak sound transmission loss and improving the dip across a wide frequency range around the stopband [48]. Additionally, in FH_S_ and FH_G_, the friction and dissipation effects of straw fibers on sound waves are more significantly exerted, so the acoustic performance of FH_S_ is better.

This section studies the effects of different filling materials on the sound insulation performance of FH and finds that straw and glazed hollow bead as filling materials significantly improve the sound insulation performance. In particular, FH_S_ shows the optimal sound insulation effect. And based on these conclusions, future experiments can consider altering the filling parameters of filling materials to determine the optimal sound insulation effect of FHs under different parameter combinations.

### 3.2. Influence of Facesheets

In practical engineering applications, paper honeycomb-core (unit) is usually used in combination with upper and lower facesheets to form an integral structure, enhancing its strength and durability. Therefore, this study further studies FHWs with paper fiber/cement facesheets. Based on the basic research in Section 3.1, it is found that straw and glazed hollow bead as filling materials are significantly better than other materials in terms of acoustic performance. Therefore, this section selects these two filling materials as representatives to study the difference in sound insulation performance between FH and FHW, that is, the influence of wallboards (paper fiber/cement facesheet) on sound insulation performance. By comparing the sound insulation performance of FHW with the previously studied FH, the effect of adding facesheets on sound insulation performance is explored.

**Figure 6:**
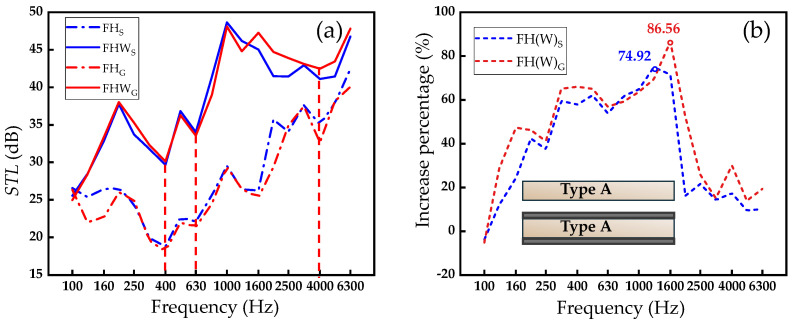
Sound insulation performance of FHWs with and without facesheets. (**a**) *STL*–frequency curve. (**b**) Improvement percentage–frequency curve achieved by adding facesheets.

Figure 6a shows the representative *STL*–frequency curves of FH_S_, FH_G_, FHW_S_, and FHW_G_. The results indicate that the acoustic performance of FHW_S_ and FHW_G_ with facesheets is significantly better than that of FH_S_ and FH_G_. Comparing FHWs with FHs, it can be observed that the addition of paper fiber/cement facesheets increases the natural frequency of the structure from around 315 Hz to approximately 400 Hz (the first red dotted line). This is because the lightweight and high-stiffness paper fiber/cement facesheets added away from the central axis significantly enhance the bending stiffness of the structure, offsetting the effect of the added mass and thus increasing the natural frequency [49]. Additionally, as the sound wave frequency increases from 100 Hz to 400 Hz (natural frequency) initially, the *STL* first increases and then decreases. This may be because, initially, the increased damping effect [47,50] and stiffness [51] from the paper fiber/cement facesheets effectively absorb and reflect the sound wave energy in this frequency range, causing the *STL* to increase with the rise in frequency. However, as the sound wave frequency continues to increase and approaches the natural frequency of 400 Hz, the resonance effect becomes stronger, resulting in a decrease in *STL*. And the turning point of the *STL* occurs around 200 Hz. After 630 Hz (the second red dotted line), the sound insulation performance of the honeycomb structure is mainly governed by the mass law. Similar to FHs, FHWs also exhibit a trough around 4000 Hz (the third red dotted line), after which the sound insulation performance continues to follow the mass law.

Figure 6b further quantifies the improvement percentage, indicating that in the frequency range of 100–6300 Hz, the *STL* of FHW_S_ and FHW_G_ is generally improved compared with FHS and FH_G_, with the maximum improvement values reaching 74.92% and 86.56%, respectively. The improvement of the sound insulation performance of FHW by facesheets may be attributed to the fact that the addition of facesheets prolongs the reflection path of sound waves [52] and utilizes the damping effect of the adhesive interface.

Based on the research on filling materials in the previous section (straw and glazed hollow bead as filling materials significantly improve the sound insulation performance), this section further finds that when paper fiber/cement facesheets are used in combination with paper honeycomb core, the sound insulation performance has been further improved.

### 3.3. Influence of Core Type

In the previous discussion on filling materials and facesheets, the core consisted of one unit. Therefore, this section will explore how multi-units (two units and three units) affect acoustic performance. In the analysis results of Section 3.1 and Section 3.2, it can be found that filling with straw and glazed hollow beads increased the *STL* by more than 50% compared to no filling in the frequency bandwidth above 2000 Hz. Furthermore, adding upper and lower paper fiber/cement facesheets further improved the sound insulation performance. These significant improvements make FHW_S_ and FHW_G_ ideal candidates for investigating the effects of core stacking on sound insulation performance.

**Figure 7:**
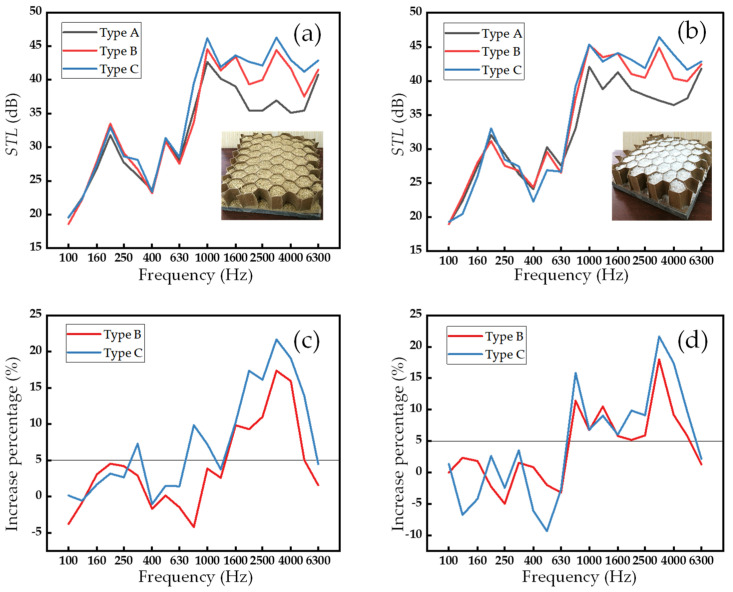
Sound insulation performance of FHWs with different core types. (**a**) *STL*–frequency curve of FHW_S_. (**b**) *STL*–frequency curve of FHW_G_. (**c**) Improvement percentage–frequency curve of FHW_S._ (**d**) Improvement percentage–frequency curve of FHW_G_.

Figure 7a,b are the *STL*–frequency curves of FHW_S_ and FHW_G_ under three different core types. In sandwich plates with different fillers, type A, type B, and type C curves show similar trends overall, and it can be roughly judged that FHWs with multi-unit superposition have a better sound insulation effect. This section also adopts a similar evaluation method as Section 3.1, but here the bandwidth is the sum of the frequency ranges where the *STL* increases by more than or equal to 5% (Figure 7c,d). This is used to calculate the relative improvement of multi-unit sound insulation compared to one-unit sound insulation. For FHW_S_ and FHW_G_ of type B, the bandwidths are 3500 and 4800 Hz, respectively. And for FHW_S_ and FHW_G_ of type C, the bandwidth increases to 5370 and 5400 Hz, respectively. This demonstrates that increasing the number of core units enhances the sound insulation performance of FHWs. The likely reason is that the increase in the overall mass and stiffness of the structure leads to improved sound insulation performance [53,54]. From a structural point of view, the additional intermediate reflective surface paper layer also increases the stiffness of the structure, and the interaction between multi-units in the core may introduce additional internal coupling effects, thereby producing a more effective suppression of acoustic vibrations. Moreover, it can be found that in the low-frequency region (100–630 Hz), the type of core (number of units) has no significant effect on its sound insulation performance, but in the mid- and high-frequency regions (630–6300 Hz), there is a large difference. This may be because the more stable internal structural system formed by the winding and interweaving of straw fibers inside the core, along with the more stable stacking of glazed hollow bead as a harder solid, provides a physical structural stability basis for reflecting high-frequency sound waves but has little effect on low-frequency sound waves.

By studying the influence of the multi-unit structure on the sound insulation performance of FHW, it was found that increasing the number of core unit can significantly improve the sound insulation performance, especially in the mid-to-high frequency region.

In summary, selecting appropriate filling materials (such as straw or glazed hollow bead) and increasing the number of core units, combined with the use of upper and lower facesheets, can significantly optimize the sound insulation performance of honeycomb structure wallboards. This finding provides an important basis for further optimizing the design of honeycomb structure wallboards and helps to improve the sound insulation effect of building structures in actual projects.

## 4. Conclusions

This study focuses on the widely adopted honeycomb structure within the industry. Based on existing conclusions about the impact of honeycomb structure dimensions on sound insulation performance, this study selects the most commonly used paper honeycomb available on the market and conducts filling experiments while keeping the honeycomb structure fixed. It also conducts an in-depth analysis of the impact of filling materials, facesheet configurations, and core types on the sound insulation performance of FHWs. The main conclusions are as follows:

(1) Acoustic impact and mechanism of filling materials: Straw filling and glazed hollow bead filling significantly enhance the sound insulation of FHs. Specifically, the bandwidth with a sound insulation improvement of more than 50% is 2310 Hz for FH_S_ and 2300 Hz for FH_G_, which is significantly higher than the 560 Hz for FH_F_. The superior performance is due to the high damping coefficient of straw and glazed hollow bead, making them more effective at absorbing and reflecting sound waves.
(2) Improvement of acoustic performance of structural facesheets: Adding facesheets not only aligns more closely with practical engineering applications but also significantly improves the sound insulation performance of FHW. For instance, the *STL* of FHW_S_ and FHW_G_ is enhanced across the 100–6300 Hz range with maximum improvements of 74.92% and 86.56%, respectively, compared to FH_S_ and FH_G_ without facesheets. This enhancement is attributed to the increased reflection paths and the damping effect of the adhesive interface.
(3) Acoustic optimization of the core structure: Increasing the number of core units enhances the acoustic performance of FHW. In FHW_S_, the bandwidth with a sound insulation improvement of over 5% increases from 3500 Hz for two units compared to one unit to 5400 Hz when the core units are increased to three. The improvement in the sound insulation performance of the structure is due to the increased mass and multi-layered structure, which provide more opportunities for scattering and absorbing sound waves.
(4) This experimental study enriches the application of honeycomb plates in sound insulation by introducing paper fiber/cement facesheet straws. The results indicate that the sound insulation performance and strength have been improved, making these plates more promising for applications in non-load-bearing structures. Besides acoustic benefits, straw is a low-cost, environmentally friendly, renewable resource that reduces waste and lowers carbon emissions, making it a green building material. This research provides valuable guidance for promoting filled honeycomb-core sound insulation wallboards in green buildings.

## Figures and Tables

**Table 1:** Material properties, manufacturers, and photographs of filling materials.

Material Name	Size (mm)	Density (kg·m^−3^)	Elastic Modulus (GPa)	Damping Coefficient (N·s·m^−1^)	Poisson’s Ratio	Manufacturer	Photograph
Sun-dried rice straw chaff	1~5	50~150	0.5~3.5	0.02~0.1	0.2~0.4	Rural Area (Lianyungang City, Jiangsu Province, China)	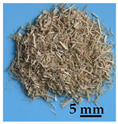
Glazed hollow bead	1~5	50~200	0.3~1.5	0.01~0.05	0.2~0.3	Zhongsen Perlite Application Co., Ltd. (Xinyang, China)	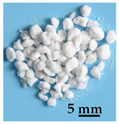
Polystyrene foam particles	3~5	10~50	0.03~0.1	0.005~0.02	0.3~0.35	Yishi Yijia Composite Material Products Co., Ltd. (Guangzhou, China)	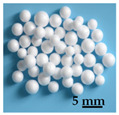

## Data Availability

Data can be made available upon request by contacting chenjpaper@yahoo.co.jp.
